# Development of a Duplex TaqMan Real-Time Polymerase Chain Reaction for Accurate Identification and Quantification of *Salmonella* Enteritidis from Laboratory Samples and Contaminated Chicken Eggs

**DOI:** 10.3390/foods11050742

**Published:** 2022-03-03

**Authors:** Dan Xiong, Yi Zhou, Li Song, Bowen Liu, Chelea Matchawe, Xiang Chen, Roger Pelle, Xinan Jiao, Zhiming Pan

**Affiliations:** 1 Jiangsu Key Laboratory of Zoonosis, Yangzhou University, Yangzhou 225009, China; xiongdan@yzu.edu.cn (D.X.); YiZhou19962020@163.com (Y.Z.); lisong@yzu.edu.cn (L.S.); lbw132101110@126.com (B.L.); chenxiang@yzu.edu.cn (X.C.); zmpan@yzu.edu.cn (Z.P.); 2 Jiangsu Co-Innovation Center for Prevention and Control of Important Animal Infectious Diseases and Zoonoses, Yangzhou University, Yangzhou 225009, China; 3 Joint International Research Laboratory of Agriculture and Agri-Product Safety of the Ministry of Education, Yangzhou University, Yangzhou 225009, China; 4 Key Laboratory of Prevention and Control of Biological Hazard Factors (Animal Origin) for Agrifood Safety and Quality, Ministry of Agriculture of China, Yangzhou University, Yangzhou 225009, China; 5 Biosciences Eastern and Central Africa-International Livestock Research Institute (BecA-ILRI) Hub, Nairobi 00100, Kenya; matchawe@yahoo.com (C.M.); r.pelle@cgiar.org (R.P.); 6 Institute of Medical Research and Medicinal Plants Studies, Yaounde 4123, Cameroon

**Keywords:** *Salmonella* *enteritidis*, duplex TaqMan real-time PCR, accurate identification, quantification, chicken egg, MPN-qPCR-SIT

## Abstract

*Salmonella* *enteritidis* is a major causative agent of foodborne illnesses worldwide. As the traditional serotyping and quantification methods are labor-intensive, time-consuming, and expensive, faster and more convenient molecular diagnostic methods are needed. In this study, we developed and validated a rapid duplex TaqMan real-time polymerase chain reaction (PCR) for the accurate identification and quantification of *S. enteritidis*. The primers and TaqMan probes were designed based on the *S. enteritidis*-specific gene *lygD* and the *Salmonella* genus-specific gene *invA*. The melt curve and gel electrophoresis analysis showed that the designed primers had potent specificity for the amplification of *lygD* and *invA*. The duplex real-time PCR specifically identified *S. enteritidis* from a panel of 40 *Salmonella* strains that represented 29 serovars and 12 non-*Salmonella* organisms. The duplex real-time PCR assay detected four copies of *S. enteritidis* DNA per reaction. The intra- and inter- assays indicated a high degree of reproducibility. The real-time PCR could accurately detect and quantify *S. enteritidis* in chicken organs after *Salmonella* infection. Furthermore, the assay identified 100% of the *S. enteritidis* and *Salmonella* genus isolates from chicken egg samples with superior sensitivity after 6 h of pre-enrichment compared to the traditional culture method. Additionally, the most-probable-number (MPN) combined with qPCR and a shortened incubation time (MPN-qPCR-SIT) method was developed for the population determination of *S. enteritidis* and compared with various enumeration methods. Thus, we have established and validated a new duplex real-time PCR assay and MPN-qPCR-SIT method for the accurate detection and quantification of *S. enteritidis*, which could contribute to meeting the need for fast detection and identification in prevention and control measures for food safety.

## 1. Introduction

*Salmonella* is one of the most important food-borne pathogens, and can cause severe enteritis worldwide. Almost 1.3 billion cases of human salmonellosis occur and approximately 3.5 million patients die as a result of the disease annually [1]. Most of these cases result from the uptake of *Salmonella*-contaminated food such as pork, poultry, and eggs [2]. Human salmonellosis symptoms include gastroenteritis, fever, diarrhea, and serious systemic infections that may cause hospitalization [3].

*S. enteritidis* has the ability to survive in the egg white and efficiently contaminate eggs [4], which leads to significant economic and health burdens worldwide [5,6]. Raw or undercooked poultry meat and eggs are foods with a high risk of leading to human salmonellosis, and several outbreaks due to these foods have been reported [7]. Although more than 2600 *Salmonella* serovars exist, *S. enteritidis* is one of the most important agents resulting in severe infection [8,9], amounting to over 60% of human salmonellosis cases in Europe [10].

Traditional *Salmonella* serotyping is conducted according to the Kauffman–White scheme by using the specific antisera for the bacterial surface O and H antigens [11]. Despite its widespread use, the traditional culture-based method is relatively expensive, labor-intensive, and time-consuming, as it often involves several enrichment steps followed by biochemical or serological confirmation, taking 3–5 days [12,13]. Since an accurate surveillance method is important for controlling the spread of salmonellosis, developing a reliable method is critical for the identification of prevalent serovars isolated from contaminated foods.

Most of the foodborne outbreaks come from animal origin food, including beef meat, poultry, eggs, and milk products, which may be contaminated by multiple pathogens including *Salmonella enterica* [14]. Fresh-cut produce is at great risk of *Salmonella* contamination, and a one-step quantitative real-time polymerase chain reaction (qRT-PCR) assay has been used to detect *Salmonella* in fresh-cut vegetables [15]. A fluorescent biosensor with multiple fluorescent signal amplification based on a streptavidin biotin system was established to detect *Salmonella* in milk. The detection limit was ten times better than that of the conventional sandwich enzyme linked immunosorbent assay (ELISA) [16]. A sensitive immunosensor was successfully constructed based on a Fe_3_O_4_ graphene nanocomposite to detect *Salmonella*. The constructed immunosensor exhibited acceptable selectivity and reproducibility for detecting *Salmonella* in milk [17]; however, a convenient method for rapid, accurate, and quantitative identification of *Salmonella* is urgently needed.

Rapid molecular methods to identify *Salmonella* serovars, especially clinically important serovars, could promote routine surveillance and therefore, public health. Of these methods, polymerase chain reaction (PCR) has been widely studied because it has high throughput, and is rapid, facile, highly sensitive, and highly specific [18,19]. Unlike traditional PCR, real-time PCR methods have gained more attention recently because the results are monitored in real time. Therefore, no other analyses or assays, such as gel electrophoresis, are needed for conformation of the specific pathogens, and the data can be analyzed quantitatively. More importantly, real-time PCR assays provide high quality quantitative data for a specific pathogen in food products [20]. The most commonly targeted *Salmonella* gene is *invA* (invasion protein gene), which is necessary for virulence and encodes a membrane protein of the type III secretion system [21,22,23,24]. The *invA* gene has been widely used for the identification of the *Salmonella* genus and used as the internal control reference [25]. Previously we found that the *Salmonella* gene *lygD* shares 98–100% similarity in nucleotide sequences and only exists in *S. enteritidis* [26,27]. Thus, the *lygD* gene is a desirable candidate for the specific identification of *S. enteritidis*.

The most probable number (MPN) method is based on decimal dilutions and has been used for the quantification of bacterial contamination in low levels. In a ten-fold dilution MPN, the bacteria number increases in each well following the incubation. Eventually, bacteria-positive wells can be observed, even if at least one bacteria cell exists before incubation. The calculation of the number of bacteria was based on the standard MPN table [28]. This method has been widely applied for the quantification of low levels of *Salmonella* and *Vibrio parahaemolyticus* in samples [29,30,31]. The combination of qPCR and MPN methods has also been used for the quantification of *Listeria monocytogenes* and *Vibrio parahaemolyticus*, and the qPCR was used as a confirmation step following MPN assays by a shortened incubation time (SIT) to determine positive/negative results [32,33,34].

In the present study, we selected the *lygD* and *invA* genes for developing a fast and accurate TaqMan real-time PCR assay for the timely identification and quantification of *S. enteritidis*. We determined the specificity, sensitivity and its accuracy of the PCR method by comparing it with that of the traditional culture method. The developed method was applied for the identification of *S. enteritidis* in chicken organs or directly contaminated chicken egg samples.

## 2. Materials and Methods

### 2.1. Bacterial Strains

Forty strains of *Salmonella* (29 serovars) and 12 non-*Salmonella* organisms were used in this study. They were either commercially obtained, or previously isolated after routine monitoring (Table 1). The strains were used to test the sensitivity and specificity of the duplex real-time PCR method.

### 2.2. Bacterial Growth and Genomic DNA Extraction

Bacterial genomic DNA was prepared as previously described [27]. In brief, all bacteria used in this study were inoculated in either brain heart infusion broth (Becton, Dickinson and Company, Sparks, MD, USA) or Luria–Bertani (LB) broth (Oxoid, Basingstoke, Hampshire, UK) at 37 °C overnight in a shaker incubator. DNA extracts were prepared from 1.0 mL of overnight cultures of the test isolates using a TIANamp Bacterial DNA kit (TianGen Biotech Co. Ltd., Beijing, China). The DNA concentration was determined using a NanoDrop ND-1000 (Thermo Scientific, Wilmington, DE, USA) spectrophotometer. The number of genomic DNA copies present in the bacterial strains was determined based on the online URI Genomics & Sequencing Center copy number calculator for double-stranded DNA (http://cels.uri.edu/gsc/cndna.html, accessed on 9 June 2021). DNA extracts were stored at −20 °C until use.

### 2.3. PCR Primer Pairs and TaqMan Probes

The duplex TaqMan real-time PCR was designed by targeting the *S. enteritidis*-specific *lygD* gene and the *Salmonella* genus-specific *invA* gene. The different primer/probe sets were designed based on sequence data available at the National Center for Biotechnology Information databases using Primer 3 software (http://bioinfo.ut.ee/primer3-0.4.0/, accessed on 16 May 2021; [38]) (Table 2). The specific probes and primers were synthesized by Takara (Dalian, China).

### 2.4. Conventional PCR

The conventional PCR assay was performed at a final volume of 20 μL including 10 μL of 2× Taq Master Mix (Vazyme, Nanjing, China), 0.5 μM of the *lygD* or *invA* F/R primers (Table 2), and the specified amount of DNA. The PCR amplification was carried out using a T100 Thermal Cycler (Bio-Rad, Hercules, CA, USA) with the following protocol: initial denaturation at 94 °C for 3 min, 30 cycles of 94 °C for 30 s, 60 °C for 30 s, 72 °C for 30 s, and a final extension of 72 °C for 10 min. The PCR products were run on 1% agarose gels.

### 2.5. SYBR-Based qRT-PCR

The melt curves for the *lygD* and *invA* products were collected by the SYBR-based qRT-PCR using serial concentrations of positive standard samples. The qRT-PCR was performed using ABI 7500 real-time instrument (Applied Biosystems, Carlsbad, CA, USA). The 20 μL PCR reactions contained 10 μL of 2× SYBR Green I Master Mix (Vazyme, Nanjing, China), 0.3 μM *lygD* or *invA* forward and reverse primers, nuclease-free water and the specified amount of DNA. The PCR profile consisted of initial denaturation at 95 °C for 5 min, followed by 40 cycles of 95 °C for 5 s, and 60 °C for 34 s. The melting curve analysis consisted of 1 cycle at 95 °C for 15 s and then 60 °C for 1 min, followed by a continuous increase of temperature to 95 °C at a rate of 0.5 °C/s. The fluorescence signal was monitored continuously and plotted against the temperature. The resulting PCR amplicons were directly visualized by 1% agarose gel electrophoresis.

### 2.6. Duplex TaqMan Real-Time PCR System

To identify *S. enteritidis*, the designed duplex PCR assay exploited the specific primers and probes for the *lygD* and *invA*. Real-time duplex PCR was conducted on an ABI 7500 instrument (Applied Biosystems, Foster, CA, USA) with the Premix Ex Taq Master kit (Takara). The reaction system (25 μL) contained DNA template (2.5 μL), 2× Premix Ex Taq Master (12.5 μL), 240 nM *lygD* forward and reverse primers (0.6 μL each), 100 nM probe (0.25 μL), 200 nM *invA* forward and reverse primers (0.5 μL each), 80 nM probe (0.2 μL), and ROX Reference Dye II (0.25 μL). The control tubes used the same mixture, without any DNA template. The real-time PCR reactions were performed for 30 s at 95 °C, followed by 40 cycles of denaturation at 95 °C for 5 s and annealing/extension at 60 °C for 34 s. The fluorescence was collected during the extension step of each cycle.

### 2.7. Specificity of the Duplex Real-Time PCR

The specificity of the two pairs of primers and probes in the duplex PCR method was determined from 40 different *Salmonella* strains and 12 other non-*Salmonella* strains using 10^5^ copies of genomic DNA for each strain listed in Table 1.

### 2.8. Standard Curve and Detection Limit of the Duplex Real-Time PCR

The standard curve and detection limit of the duplex real-time PCR were evaluated using *S. enteritidis* C50041. Bacterial counts were verified based on 10-fold dilutions (10^1^–10^8^ dilutions) and a traditional plate counting assay. Genomic DNA was prepared from decimally diluted samples and amplified by the duplex real-time PCR. Ct values of each dilution were obtained and plotted against log10 colony-forming units (CFU). The linear ranges were assessed using the established standard curves.

### 2.9. Evaluation of the Reproducibility of the Method in Detecting S. enteritidis

Reproducibility for the identification and quantification of *S. enteritidis* was conducted on six standard samples of *S. enteritidis*. Different concentrations of *S. enteritidis* (4 × 10^1^~4 × 10^6^ copies/μL) were used as the templates for TaqMan real-time PCR assay. The intra-batch reproducibility experiment was conducted with three repetitions of the template in one TaqMan real-time assay. The inter-batch reproducibility experiment was conducted by measuring the same template three times by three operators independently. Finally, the coefficient of variation (CV) of the Ct values was determined based on the intra-assay or inter-assay results, so as to evaluate the reproducibility of the method.

### 2.10. Real-Time PCR for Quantification of S. enteritidis in Organs in a Chicken Model

Two-week-old specific-pathogen free (SPF) white Leghorn chickens were purchased from the poultry institute, at the Shandong academy of agricultural science. All chickens were housed in a room with controlled ventilation, light, and temperature. The procedures described in this study were approved by the Committee on the Ethics of Animal Experiments of Yangzhou University (Approval ID: SYXK (Su) 2017-0044). For oral infections, chickens were fasted overnight and subsequently inoculated orally with 1 × 10^6^ CFU of bacteria in 0.2 mL phosphate-buffered saline (PBS). They were sacrificed three days post-infection, and the spleens and livers were collected to calculate the bacterial burden. The tissues were harvested in 2 mL pre-weighed tubes containing 0.1 mL PBS and weighed before homogenization using the Precellys 24 homogenizer (Rockville, MD, USA). The homogenate dilutions (100 μL each) were used for plate counting on LB agar. The data represent the number of CFU/mL of the organs. The 10^−3^ dilutions were incubated at 100 °C for 15 min and placed on ice immediately. The tubes were centrifuged for 5 min at 12,000 rpm at 4 °C. An aliquot of the supernatant (2 μL) was served as the template DNA in the duplex TaqMan real-time PCR.

### 2.11. Real-Time PCR for the Detection of S. enteritidis in Clinical Chicken Eggs

The sensitivity and accuracy of the real-time PCR in *S. enteritidis* detection in clinical dead egg samples was assessed using 70 samples from a chicken farm and compared to a traditional serotyping method. Samples from the chicken farm were collected as previously described [36,39]. Pre-PCR samples were prepared with a pre-enrichment step in buffered peptone water (BPW), followed by DNA extraction. In brief, 45 mL of BPW was added to the livers of the chicken eggs and incubated at 37 °C at 100 rpm for 6 h. The genomic DNA was extracted from one milliliter of each pre-enriched sample, and subsequently used for the real-time PCR.

### 2.12. Traditional Serotyping of Salmonella Isolates from Clinical Samples

The traditional serotyping method was conducted by incubating the sample enrichments as prepared above for an additional 18 h. Then, 0.1 mL of the broth culture was subcultured in 10 mL Rappaport–Vassiliadis (RV) enrichment broth (Difco, BD) at 42 °C for 24 h. One loopful of each RV broth culture was streaked on to xylose lysine tergitol 4 (Difco, BD) agar plates, and then incubated at 37 °C for 24–48 h. The presumptive *Salmonella* colony was picked from each plate and biochemically confirmed using an API-20E test kit (bioMérieux, Marcyl’Etoile, France). All *Salmonella* isolates from clinical contaminated samples were serotyped following the White–Kauffmann–Le Minor scheme based on agglutination with O- and H-antigen-specific sera (Tianrun Bio-Pharmaceutical, Ningbo, China). All samples were examined by conventional microbiological methods and compared with the duplex TaqMan real-time PCR in a blind manner.

### 2.13. Preparation of S. enteritidis Cell Suspension and Enumeration Methods

One colony of *S. enteritidis* on LB agar was expanded by growing cultures overnight at 37 °C. The *S. enteritidis* cells were harvested by centrifugation at 12,000 rpm for 2 min. The cell pellets were washed twice and resuspended in PBS. Serial dilutions of the cell suspension were used to obtain incrementally different bacterial concentrations. The populations of *S. enteritidis* were evaluated by different enumeration methods including traditional plating, traditional MPN, TaqMan real-time PCR, and the MPN-qPCR-SIT established in this study.

### 2.14. Traditional Plating and Traditional MPN Methods

Undiluted and serially diluted cell suspensions (100 µL of each sample) as prepared above were plated on LB agar, and subsequently incubated at 37 °C overnight. The traditional plating method was conducted by counting colonies for the determination of the *S. enteritidis* population. The conventional MPN method was conducted as previously described [34]. In brief, the assay was conducted using the 96-well sterile microtiter plates (Jet Biofil, Guangzhou, China). The 10 prepared samples containing different populations of *Salmonella* cells were serially diluted. A miniature of the three tube MPN assay was conducted by dispensing 200 µL of each diluent to three wells. The plates with the cell suspensions of *S. enteritidis* were cultured at 37 °C for 24 h. The negative and positive results of each well were evaluated visually via turbidity. The populations of *S. enteritidis* in each sample were determined based on the number of positive wells according to the standard MPN table [28].

### 2.15. TaqMan Real-Time PCR and MPN-qPCR-SIT Methods

To increase the DNA concentration of the *Salmonella* suspensions prepared above, the genomic DNA was extracted with a final elution volume of 20 µL from one milliliter of pure cultures. Extracted DNA (5 µL) was used as the template in the TaqMan real-time PCR system. The population of *S. enteritidis* was calculated based on the established standard curve. The MPN-qPCR-SIT method was prepared with the previously described conventional MPN procedure [34]. A short incubation time of 4 h was applied for the MPN plates. Genomic DNA from each well was harvested from the enriched cultures, and the negative or positive *S. enteritidis* levels were determined by the TaqMan real-time PCR.

### 2.16. Statistical Analysis

The significances of the differences of the bacterial loads in chicken organs, as determined by duplex real-time PCR and plate counting methods, were analyzed using the Student’s *t*-test with GraphPad Prism 5.0 (San Diego, CA, USA). Scatter plots were produced to present correlative relationships of the MPN-qPCR-SIT with the other methods. The MPN-qPCR-SIT and various enumeration methods were compared through linear regression and the coefficients from the scatter plots. A Bland–Altman plot was used to evaluate the 95% agreement boundaries of the different enumeration methods. The scatter plot, Bland–Altman plot, and regression trend lines were generated by using GraphPad Prism.

## 3. Results

### 3.1. Specificity Analysis of the Primers for the Amplification of lygD and invA

The melt curves of the SYBR-based qRT-PCR were analyzed to determine the specificity of the primers. The qRT-PCR assay was conducted with different concentrations of DNA, and the results were assessed by both melt curves and agarose gel electrophoresis. As seen in Figure S1A, the melt curve data showed single peaks of 79.58 °C for *lygD* and 80.56 °C for *invA*, suggesting that the primers had good specificity for the amplification of the both genes. In addition, a direct visualization of the products of the SYBR-based qRT-PCR showed that only one specific band was observed for either *lygD* or *invA* (Figure S1B). The conventional PCR results also showed that only one specific band corresponding to either *lygD* or *invA* of *S. enteritidis* was generated, respectively (Figure S1C).

### 3.2. Specificity of the Duplex TaqMan Real-Time PCR Assay

The specificity of the real-time PCR assay for the targets was determined using 40 strains of different *Salmonella* serovars and 12 other non-*Salmonella* bacterial strains. All *Salmonella* strains tested positive for *invA*. Both *lygD* and *invA* were amplified and detected in all *S. enteritidis* strains. None of the non-*Salmonella* strains produced signals for the *lygD* or *invA* targets. This indicated that *lygD*- and *invA*-based duplex TaqMan real-time PCR detects *S. enteritidis* specifically (Table 1).

### 3.3. Standard Curves and Sensitivity of the Developed Real-Time PCR

**Figure 1:**
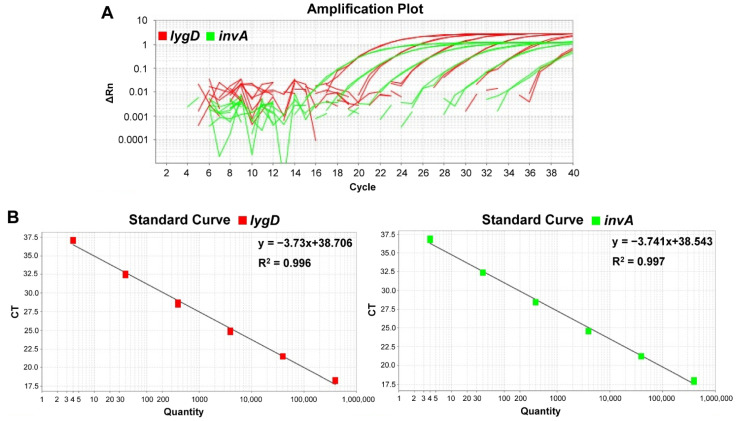
Sensitivity and standard curves of the developed duplex TaqMan real-time polymerase chain reaction (PCR) assay. The detection limit of the PCR assay was determined by testing various concentrations of *Salmonella* Enteritidis C50041 genomic DNA, ranging from 4–4 × 10^5^ copies per reaction. The PCR system contained probes and primers specific for *S. enteritidis* and *Salmonella* spp. (**A**) Amplification plots of the duplex real-time PCR. X axis—PCR cycle numbers; y axis—fluorescence intensity. (**B**) Standard curves indicating the linearity for detecting *lygD* and *invA* by real-time PCR. There was a good linear correlation between the Ct values of *lygD* and *invA* and the logarithm of the DNA copy numbers over the whole range of DNA concentration. The Ct values were plotted against the corresponding *Salmonella* cell numbers.

The detection limit of DNA concentration corresponding to the bacterial concentration was determined. Standard curves were generated by using the mean Ct values for various concentrations of *S. enteritidis* C50041 genomic DNA, ranging from 4–4 × 10^5^ copies per reaction in the real-time PCR system. A good linearity of response (R^2^ = 0.996 and 0.997) for each reaction channel (Cy5 and BHQ-2 for *lygD*, FAM and TAMRA for *invA*) was observed between the Ct values and the amount of bacterial DNA. The results indicated that the duplex TaqMan real-time PCR could successfully detect as low as four copies per reaction of *S. enteritidis* DNA (Figure 1).

### 3.4. Reproducibility of the TaqMan Real-Time PCR Assay

The duplex TaqMan real-time PCR assay produced very similar Ct values when tested on six samples of the target pathogen, *S. enteritidis*. The intra-assay CVs of the Ct values for the duplex real-time PCR ranged from 0.33% to 1.94% and 0.17% to 1.60% for *lygD* and *invA*, respectively. The inter-assay reproducibility experiments showed that the inter-assay CVs ranged from 0.80% to 2.22% and 0.54% to 2.54% for *lygD* and *invA*, respectively (Table 3). The results indicated a high degree of reproducibility of this assay.

### 3.5. Quantification of S. enteritidis in a Chicken Model by Real-Time PCR Assay

**Figure 2:**
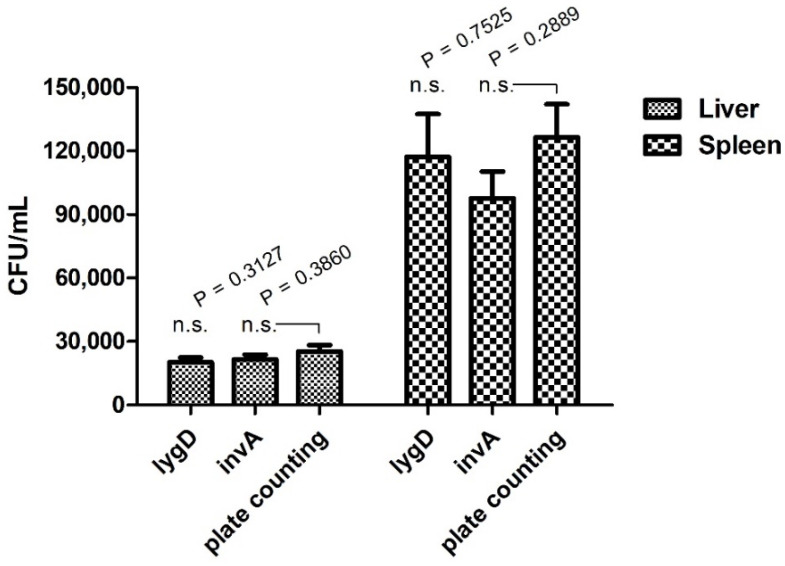
Quantification analysis of the duplex real-time PCR for *S. enteritidis* in a chicken infection model. SPF white Leghorn chickens were infected with the indicated concentrations of bacteria in 0.2 mL phosphate-buffered saline orally. The spleens and livers were collected for analysis of bacterial burden. Organs were collected and homogenized. The serial 10-fold dilutions were plated on LB agar. The data represent the number of CFU/mL in 10^−3^ dilution of organs. n.s. indicates no significant difference.

To evaluate the applicability and reliability of the assay in routine laboratories, a chicken infection model was applied. The bacterial burden in organs was determined by both the developed real-time PCR assay and plate counting methods. The results showed that *lygD* yielded Ct values of 32.36 ± 0.25 and 29.53 ± 0.40, 30 in 10−3 dilution of liver and spleen homogenates, respectively, while *invA* yielded Ct values of 32.08 ± 0.29 and 29.62 ± 0.30 in 10^−3^ dilution of liver and spleen homogenates, respectively. Of note, there was no significant difference in the quantification of *Salmonella* between the real-time PCR assay and the traditional plate counting method (Figure 2).

### 3.6. Application of the Duplex Real-Time PCR for Clinical Chicken Eggs

The established real-time PCR method was used to identify *S. enteritidis* in clinically contaminated egg samples from a chicken farm. A total of 70 egg samples were analyzed by the developed TaqMan real-time PCR method, and the results were compared with those obtained by traditional serotyping procedures. The prevalence (determined by real-time PCR) of the *Salmonella* genus and specifically, the *S. enteritidis* isolates were 42.86% (30/70) and 37.14% (26/70) for the TaqMan real-time PCR method and the traditional serotyping procedure respectively, after 6 h of non-selective pre-enrichment. Of the 70 samples tested, the traditional method detected 27 *Salmonella* isolates (38.57%, 27/70), including 23 strains of *S. enteritidis* (32.86%, 23/70) (Table 4). Of note, the PCR products of three *S. enteritidis*-positive isolates, not detected by the traditional method, were sequenced, and the results showed that all the PCR products were *lygD*- and *invA*-positive. Thus, the duplex TaqMan real-time PCR showed an improved clinical sensitivity and accuracy compared to the traditional serotyping method.

### 3.7. Traditional Serotyping of Salmonella Isolates and Biochemical Identification

The isolation of *Salmonella* from the clinical chicken egg samples was also carried out according to the traditional standard method. The serotypes of *Salmonella* isolates from the chicken farm were determined using slide agglutination assays based on the specific O and H antisera. The results showed that the *Salmonella* isolates from 70 egg samples included 23 strains of *S. enteritidis*, one strain of *S.* Weltevreden, and three strains of *S.* London. Compared with the TaqMan real-time PCR results, three isolates (A8, C3 and F11) of *S. enteritidis* were not identified by the traditional method (Table 4).

### 3.8. Comparison of MPN-qPCR-SIT with Other Enumeration Methods

The developed MPN-qPCR-SIT method for population determination of *S. enteritidis* was compared to the traditional MPN, traditional plating, and TaqMan real-time PCR (Table 5). The results showed that the MPN-qPCR-SIT method and the traditional MPN presented the similar bacterial population. The average difference between them was 0.039 log MPN/mL and the greatest difference was 0.277 log MPN/mL. The MPN-qPCR-SIT also showed similar results to the traditional plating method. All bacterial populations based on the traditional plating assay fell in the 95% confidence interval of the developed MPN-qPCR-SIT. The MPN-qPCR-SIT showed a potent detection sensitivity compared to the traditional PCR and plating methods. The newly developed MPN-qPCR-SIT method could identify low levels of *S. enteritidis* bacterial cells that could not be detected by traditional plating and PCR.

**Figure 3:**
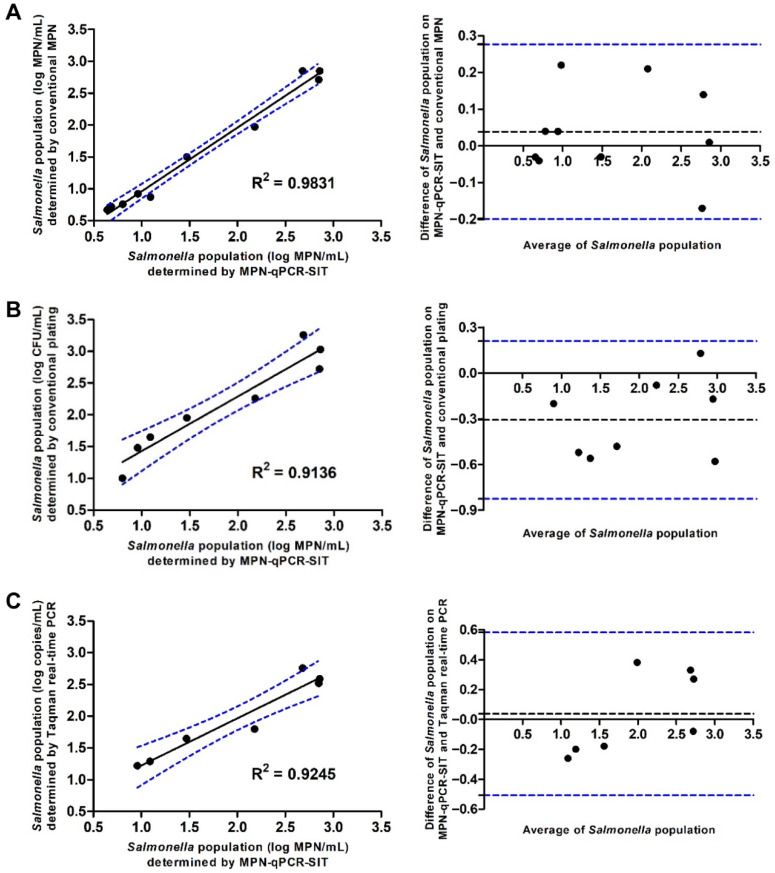
Scatter plots and Bland–Altman plots to present correlative relationships to evaluate the 95% agreement boundaries by comparison of the MPN-qPCR-SIT with other methods (*y*-axis) including (**A**) traditional MPN, (**B**) traditional plating, and (**C**) TaqMan real-time PCR for the quantification of *S. enteritidis* in bacterial cell suspensions.

To determine the efficiency of the developed MPN-qPCR-SIT, correlation analysis was conducted on the *S. enteritidis* populations. The agreement between various methods was evaluated by the Bland–Altman method. The results showed that the MPN-qPCR-SIT presented a good correlation with the traditional MPN with an R^2^ = 0.9831. The 95% limits of agreement between the two methods ranged from −0.1988 to 0.2768 log MPN/mL, indicating that the MPN-qPCR-SIT had a similar sensitivity and effectiveness when compared to the traditional MPN method (Figure 3A). Linear correlations of the MPN-qPCR-SIT were also observed with the traditional plating assay (R^2^ = 0.9136, Figure 3B) and TaqMan real-time PCR (R^2^ = 0.9245, Figure 3C). The MPN-qPCR-SIT presented the 95% agreement boundaries of −0.8242 to 0.2092 and −0.5073 to 0.5816 with the traditional plating or TaqMan PCR, respectively.

## 4. Discussion

*Salmonella* causes over one million foodborne infection cases in the US annually, and its accurate and rapid detection continues to be of important interest for both clinical diagnosis and food safety surveillance [5,40]. Compared to traditional serotyping, PCR methods are advantageous because of their simplicity and speed when many samples are to be tested for a few selected serotypes [41]. To identify *S. enteritidis* isolates directly from poultry eggs, a duplex TaqMan real-time PCR was developed and validated using two pairs of primers and TaqMan probes targeting the *lygD* and *invA* genes, which have been proven to be *S. enteritidis*-specific and *Salmonella* genus-specific, respectively [26,27]. SYBR-based qRT-PCR and conventional PCR assays showed that the designed primers had good specificity for the amplification of *lygD* and *invA*. The TaqMan real-time PCR results confirmed the specificity of the *lygD* and *invA* primer-probe sets. The present method directed toward *S. enteritidis* showed high selectivity and accuracy.

The logarithm of the *Salmonella* DNA copy numbers correlated excellently with the *lygD* and *invA* Ct values in the developed PCR assay. The correlation was linear in the entire range of DNA concentrations (4–4 × 10^5^ copies), showing that this PCR method could be used to quantify the *Salmonella* numbers present in samples. The detection limit of the duplex real-time PCR assay was 4 copies per reaction for *S. enteritidis*, which was comparable to previous studies [42], indicating that the real-time PCR method developed in this study had sufficient sensitivity to be used for diagnostic purposes and as an alternative to the traditional methods. The reproducibility of the duplex TaqMan real-time PCR assay was evaluated by testing different concentrations of the positive standard samples. The results indicated satisfactory intra- and inter-reproducibility with the CVs lower than 3%, showing better reproducibility than a previous study, where the CVs were lower than 5% [43]. Additionally, the duplex TaqMan real-time PCR provided a double check for *S. enteritidis* identification by detecting the two genes of *lygD* and *invA* simultaneously.

The current developed duplex real-time PCR method for identifying *S. enteritidis* is highly accurate and could be performed directly on 6-h pre-enriched clinical samples. The TaqMan real-time PCR method is more sensitive and saves time compared with the traditional PCR method for the identification of *Salmonella* in clinical samples [44]; however, the 6 h of non-selective pre-enrichment is necessary for the accurate identification of *S. enteritidis* isolates from the clinical samples. Thus, the pre-enrichment step should be further optimized in the future study. The PCR results of chicken egg samples demonstrated 100% sensitivity, confirming the reliability and validity of this PCR method for detecting *S. enteritidis*. The entire identification time was approximately 7 h, compared to 5–6 days for the conventional culture and serotyping method [12]. Thus, the PCR method can be a reliable and alternative rapid method for screening and quantification of *S. enteritidis* and the *Salmonella* genus in such clinical samples. Besides, the developed method in this study is not limited by the sample size. As its high accuracy and efficiency, the duplex real-time PCR could be used as an effective tool to timely identify *S. enteritidis* and *Salmonella* genus, especially in high-throughput screening situations [45].

The developed real-time PCR identified three more *S. enteritidis* isolates, indicating that it is more sensitive and accurate for the detection of *Salmonella* from clinical egg samples compared to the traditional method. Other studies also showed that qPCR is more sensitive for the detection of *Salmonella* from cattle lymph nodes samples, as compared to the culture method [18]. The present real-time PCR generated more accurate data with increased detection sensitivity for the prevalence of *Salmonella* in chicken farms. Thus, the application of the present method in food control systems, for example, at slaughterhouses, could be helpful in generating more accurate data for future surveillance of *Salmonella* serotypes of veterinary and clinical relevance.

A new enumeration method of MPN-qPCR-SIT was developed based on the established TaqMan real-time PCR. The coefficient of the MPN-qPCR-SIT presented a high level of correlation compared to the traditional MPN. Traditional MPN generally needs 1–2 days for the plates or tubes incubation [29,30], while the MPN-qPCR-SIT needs only 4 h for incubating MPN plates. Thus, the MPN-qPCR-SIT method needs a much shorter time (less than 5 h totally) than the traditional MPN. These results showed that the MPN-qPCR-SIT is an alternative method that is fast and reliable for the quantification of *S. enteritidis*. In future studies, integrating multiplex qPCR into the MPN-qPCR-SIT would be a highly useful detection technology which could identify and quantify multiple targets simultaneously within a single reaction, such as different *Salmonella* serovars or even other pathogens.

## 5. Conclusions

In summary, we developed an accurate duplex TaqMan real-time PCR method and the new MPN-qPCR-SIT enumeration method for the identification and quantification of *S. enteritidis*. The method has potent selectivity, excellent sensitivity, good reproducibility and can be easily performed. This new real-time PCR method was well applicable for the identification of *S. enteritidis* in chicken organs and clinical chicken eggs with 100% specificity in a short time, which was superior than the traditional method. Additionally, the MPN-qPCR-SIT method presented a potent detection sensitivity and showed stronger advantages than the traditional MPN method for the population determination of *S. enteritidis*. This would be useful especially in the timely detection and quantification of low levels of *Salmonella* contamination in food production systems. The validated new duplex TaqMan real-time PCR assay could be used as a convenient tool for monitoring the *Salmonella* contamination in the chicken production chain and strengthen public health through effective screening of *S. enteritidis* in clinical samples.

## Figures and Tables

**Table 1:** Different *Salmonella* serovars and other non-*Salmonella* bacteria used to determine the specificity of the established real-time PCR assay.

	Strain ^a^	Serovar/Species	Source	Duplex PCR Results	
				*lygD*	*invA*
*Salmonella*	C50041	Enteritidis	Laboratory stock	+	+
	C50336	Enteritidis	Laboratory stock	+	+
	Z11	Enteritidis	Laboratory stock	+	+
	Pi9	Enteritidis	Isolate from pig	+	+
	Ch17	Enteritidis	Isolate from chicken	+	+
	S06004	Pullorum	Laboratory stock	–	+
	6508	Pullorum	Isolate from chicken	–	+
	SG9	Gallinarum	[35]	–	+
	SL5928	Dublin	Laboratory stock	–	+
	T3	Uganda	[36]	–	+
	T9	Meleagridis	[37]	–	+
	T8	Anatis	[37]	–	+
	G2	London	[36]	–	+
	Pi16	London	Laboratory stock	–	+
	ZX	Rissen	[36]	–	+
	Y7	Derby	[36]	–	+
	Pi12	Derby	Isolate from pig	–	+
	ZHJ5	Derby	Laboratory stock	–	+
	Y8	Typhimurium	[37]	–	+
	Pi14	Typhimurium	Laboratory stock	–	+
	Pi24	Typhimurium	Laboratory stock	–	+
	C500	Choleraesuis	Laboratory stock	–	+
	ZH65	Indiana	[36]	–	+
	ZH5	Sinstorf	Laboratory stock	–	+
	ZH10	Newlands	Isolate from cattle	–	+
	ZZH24	Muenster	Laboratory stock	–	+
	ZH82	Yoruba	Isolate from pig	–	+
	G449	Dumfries	Laboratory stock	–	+
	G241	Kentucky	Laboratory stock	–	+
	G382	Agona	Laboratory stock	–	+
	ZMH35	Newport	Laboratory stock	–	+
	TJ42	Thompson	[36]	–	+
	Ch15	Thompson	Laboratory stock	–	+
	P192	Senftenberg	Laboratory stock	–	+
	G439	Blockley	Laboratory stock	–	+
	G86	Inchpark	Laboratory stock	–	+
	P122	Virchow	Laboratory stock	–	+
	P74	Farsta	Laboratory stock	–	+
	G85	Dabou	Laboratory stock	–	+
	GS3	Potsdam	Laboratory stock	–	+
Non-*Salmonella*	H37Rv	*Mycobacterium tuberculosis*	ATCC 27294	–	–
	11168	*Campylobacter jejuni*	ATCC 700819	–	–
	TH5	*Campylobacter jejuni*	Isolate from chicken	–	–
	cj18	*Campylobacter jejuni*	Laboratory stock	–	–
	S19	*Brucella abortus*	Laboratory stock	–	–
	51592	*Shigella flexneri*	Laboratory stock	–	–
	EGDe	*Listeria monocytogenes*	ATCC BAA-679	–	–
	LM23	*Listeria monocytogenes*	Laboratory stock	–	–
	1314	*Escherichia coli*	Isolate from chicken	–	–
	E10	*Escherichia coli*	Laboratory stock	–	–
	8-1-6	*Escherichia coli*	Isolate from chicken	–	–
	27217	*Staphylococcus aureus*	ATCC 27217	–	–

^a^ Equal concentrations (0.2 ng/μL) of genomic DNA from each strain were tested in the TaqMan real-time PCR assay.

**Table 2:** Primers and TaqMan probes for the two genes used in the duplex real-time PCR.

Gene	Primer Name	Sequence (5′-3′) ^a^	Amplicon Size (bp)	Location
*lygD*	*lygD*-F	CTTTCTCAGATTCAGGGAGTATATCA	111	CP013097.1 1469293–1469403
	*lygD*-R	GTTCTTCTGGTACTTACGATGACAAC		
	*lygD*-P	Cy5-CCTGTTGTCTGCTCACCATTCGCC-BHQ2		
*invA*	*invA*-F	GCGTTCTGAACCTTTGGTAATAA	104	CP013097.1 2915046–2915149
	*invA*-R	CGTTCGGGCAATTCGTTA		
	*invA*-P	FAM-TGGCGGTGGGTTTTGTTGTCTTCT-TAMRA		

^a^ Cy5—cyanine dye 5; BHQ2—black hole quencher 2; FAM—fluorescein amidite; TAMRA—tetramethylrhodamine.

**Table 3:** Intra- and inter-assay reproducibility results of the developed TaqMan real-time PCR assay.

Concentration of Template (Copies/μL)	Intra-Assay Reproducibility				Inter-Assay Reproducibility			
	Mean Ct ± SD		CV (%)		Mean Ct ± SD		CV (%)	
	*lygD*	*invA*	*lygD*	*invA*	*lygD*	*invA*	*lygD*	*invA*
4 × 10^6^	14.97 ± 0.2177	14.59 ± 0.2059	1.45	1.41	14.75 ± 0.3277	14.43 ± 0.3670	2.22	2.54
4 × 10^5^	18.24 ± 0.2303	17.97 ± 0.2878	1.26	1.60	18.13 ± 0.3571	17.74 ± 0.3541	1.97	2.00
4 × 10^4^	21.68 ± 0.4199	21.21 ± 0.0367	1.94	0.17	21.63 ± 0.1721	21.12 ± 0.2388	0.80	1.13
4 × 10^3^	25.55 ± 0.1740	24.79 ± 0.0759	0.68	0.31	25.32 ± 0.3970	24.62 ± 0.1324	1.57	0.54
4 × 10^2^	29.59 ± 0.0991	29.16 ± 0.1973	0.33	0.68	29.04 ± 0.4371	28.91 ± 0.4013	1.51	1.39
4 × 10^1^	33.19 ± 0.3454	32.74 ± 0.1991	1.04	0.61	32.90 ± 0.2988	32.65 ± 0.2808	0.91	0.86

**Table 4:** Identification of *S. enteritidis* and *Salmonella* genus from the contaminated chicken eggs.

Sample	Real-Time PCR		Traditional Serotyping	Sample	Real-Time PCR		Traditional Serotyping
	lygD	invA			lygD	invA	
A1	+	+	SE	C12	–	+	SL
A2	–	–	–	C13	–	–	–
A3	+	+	SE	C14	–	–	–
A4	+	+	SE	E1	–	–	–
A5	+	+	SE	E2	+	+	SE
A6	+	+	SE	E3	–	–	–
A7	+	+	SE	E4	–	–	–
A8	+	+	–	E5	–	–	–
A9	+	+	SE	E6	–	–	–
A10	+	+	SE	E7	–	–	–
A11	+	+	SE	E8	–	–	–
A12	+	+	SE	E9	+	+	SE
A13	+	+	SE	E10	+	+	SE
B1	–	–	–	E11	–	–	–
B2	–	–	–	E12	–	+	SL
B3	–	–	–	E13	–	–	–
B4	+	+	SE	E14	+	+	SE
B5	–	–	–	E15	+	+	SE
B6	–	–	–	E16	–	–	–
B7	+	+	SE	F1	–	–	–
B8	–	–	–	F2	–	–	–
B9	+	+	SE	F3	–	–	–
B10	–	–	–	F4	–	–	–
B11	–	–	–	F5	+	+	SE
B12	–	–	–	F6	–	–	–
C1	–	–	–	F7	–	–	–
C2	–	–	–	F8	–	–	–
C3	+	+	–	F9	–	–	–
C4	–	–	–	F10	–	–	–
C5	–	+	SW	F11	+	+	–
C6	–	–	–	F12	–	–	–
C7	–	–	–	F13	–	–	–
C8	+	+	SE	F14	–	+	SL
C9	+	+	SE	F15	–	–	–
C10	–	–	–	Total	26/70	30/70	23/70 (SE) 1/70 (SW) 3/70 (SL)
C11	+	+	SE				

SE, *S. enteritidis*; SW: *S.* Weltevreden; SL, *S.* London.

**Table 5:** Population determination of *S. enteritidis* (log MPN/mL or log CFU/mL) by different methods including the traditional plating, traditional MPN, TaqMan real-time PCR, and the MPN-qPCR-SIT.

Trial	MPN-qPCR-SIT (log MPN/mL)			Traditional MPN (log MPN/mL)			Traditional Plating (log CFU/mL)	Real-Time PCR (log Copies/mL)
	MPN	Lower Limit of the 95% CI	Upper Limit of the 95% CI	MPN	Lower Limit of the 95% CI	Upper Limit of the 95% CI		
1	0.64	−1.33	2.60	0.67	−1.74	3.08	ND	ND
2	0.68	−0.79	2.14	0.72	−1.25	2.68	ND	ND
3	0.80	−2.25	3.85	0.76	−1.78	3.30	1.00	ND
4	0.96	−0.13	2.04	0.92	0.34	1.49	1.48	1.22
5	1.09	−1.65	3.82	0.87	−3.07	4.81	1.65	1.29
6	1.47	0.07	2.87	1.50	−0.22	3.21	1.95	1.65
7	2.18	−0.43	4.78	1.97	1.97	1.97	2.26	1.80
8	2.85	0.44	5.26	2.71	−1.48	6.90	2.72	2.52
9	2.86	0.50	5.21	2.85	0.44	5.26	3.03	2.59
10	2.68	−1.89	7.25	2.85	0.44	5.26	3.26	2.76

ND—not detected.

## Data Availability

The data that support the findings of this study are available from the corresponding author upon reasonable request.

## Supplementary Materials

The following are available online at: https://www.mdpi.com/article/10.3390/foods11050742/s1, Figure S1: SYBR-based qRT-PCR and conventional PCR assays using ten-fold serially diluted DNA of *S. enteritidis* C50041.
