# Supplementary material for: Development of a Duplex TaqMan Real-Time Polymerase Chain Reaction for Accurate Identification and Quantification of Salmonella Enteritidis from Laboratory Samples and Contaminated Chicken Eggs
Source: Foods. 2022 Mar 3;11(5):742. doi: 10.3390/foods11050742 (PMC8909838; doi:10.3390/foods11050742)
Supplement: Supplementary file 1 [file foods-11-00742-s001.zip › foods-1587564-supplementary.pdf]

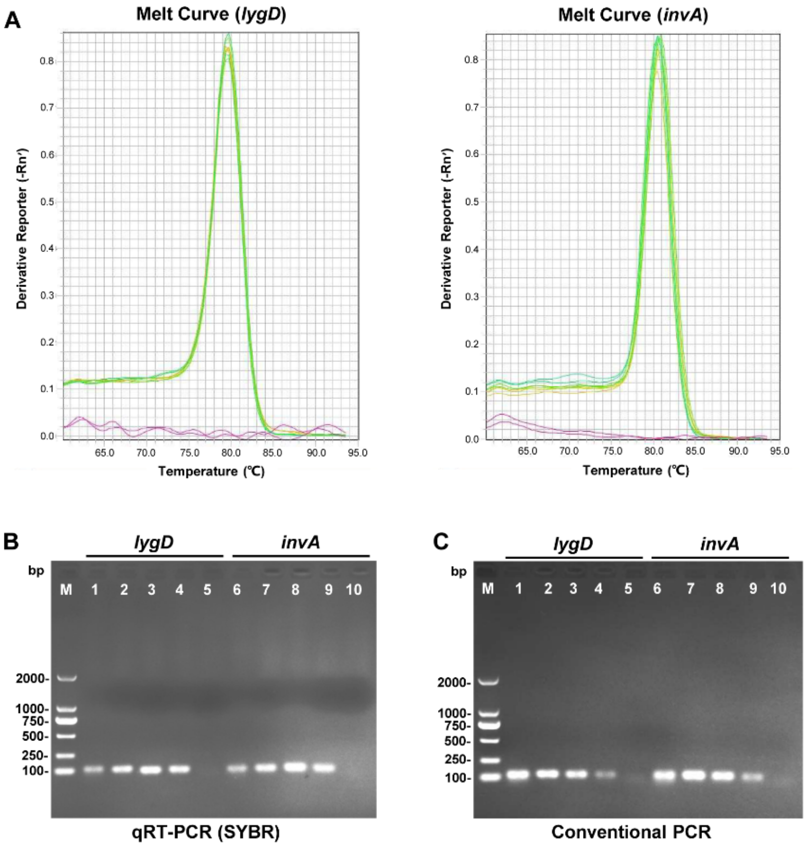

Figure S1. SYBR-based qRT-PCR and conventional PCR assays using ten-fold serially diluted DNA of *S.* Enteritidis C50041. (**A**) Melt curve analysis of SYBR green-based qRT-PCR for *lygD* and *invA*. (**B**) Agarose gel electrophoresis of SYBR green-based qRT-PCR products. (**C**) Agarose gel electrophoresis of conventional PCR products. M, DL2000 DNA marker; Lanes 1-4 and 6-9, serial dilutions (0.2~200 ng/μL) of the positive DNA; Lanes 5 and 10, no template control.
